# Data on thermal conductivity, water vapour permeability and water absorption of a cementitious mortar containing end-of-waste plastic aggregates

**DOI:** 10.1016/j.dib.2018.03.128

**Published:** 2018-03-31

**Authors:** Luciano Di Maio, Bartolomeo Coppola, Luc Courard, Frédéric Michel, Loredana Incarnato, Paola Scarfato

**Affiliations:** a University of Salerno, Department of Industrial Engineering, Via Giovanni Paolo II n. 132, 84084 Fisciano, SA, Italy; b University of Liège, ArGEnCo Department, Quartier Polytech 1, Allée de la découverte 9, B-4000 Liège, Belgium

## Abstract

The data presented in this article are related to the research article entitled “Hygro-thermal and durability properties of a lightweight mortar made with foamed plastic waste aggregates” (Coppola et al., 2018) [1]. This article focuses the attention on thermal conductivity, water vapour permeability and water absorption of a lightweight cementitious mortar containing foamed end-of-waste plastic aggregates, produced via foam extrusion process [2]. Thermal conductivity, water vapour permeability and water absorption data are made available to allow comparison and/or extend the analysis. Experimental investigations showed that the presence of plastic aggregates decreased thermal conductivity, water vapour resistance and capillary water absorption.

**Specifications table**

**Table:** 

Subject area	*Engineering, Materials Science*
More specific subject area	*Construction and waste materials*
Type of data	*Text file, tables, figures*
How data was acquired	*Laboratory balance(OHAUS, Adventurer Pro); digital caliper (MITUTOYO); GHP apparatus (experimental apparatus*[10]*); environmental chamber.*
Data format	*Raw, analyzed*
Experimental factors	*Mortar samples were prepared according EN 196-1*[3]*replacing natural silica sand (particle size distribution 0/2* *mm) with three artificial aggregates volume fractions (10, 25 and 50%). Plastic aggregates were produced according to a procedure described in*[2]*. Depending on the test, mortar samples were prepared, conditioned and tested according the properly European standard.*
Experimental features	*Data were acquired according different European standards (EN 196-1, EN 12390-7, EN 1015-15, EN 1015-19, EN 12664, EN 12667)*[3], [5], [6], [7], [8], [9]
Data source location	*Fisciano (SA), Italy, 40°46'18" N, 14°47'40" E;*
	*Sart-Tilman, Liège, Belgium, 50°35'28" N, 5°34'16" E*
Data accessibility	*Data are within this article*

**Value of the data**

• The data indicate the suitability of end-of-waste plastic aggregates for producing a lightweight mortar.
• The data can be used as comparison by researchers that are investigating lightweight mortars containing different plastic aggregates.
• The provided data can be used for validation or calibration of analytical models.
• The experimental data reported a reduced thermal conductivity, water vapour resistance and capillary absorbed water at increasing plastic aggregates content.
• Data can be further enriched using other characterization techniques.

## 1 Data

**Fig. 1:**
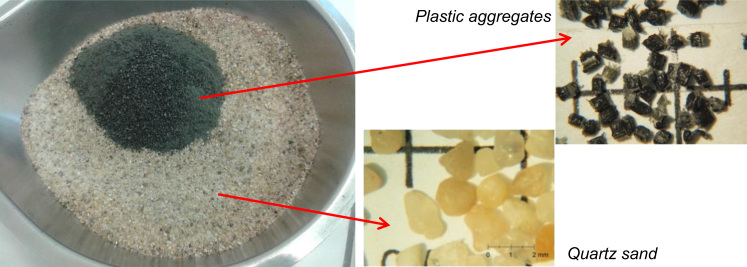
Plastic and quartz aggregates used in this research.

In this dataset are listed the results of a study concerning the use of plastic aggregates (prepared according to the procedure described in Ref. [2]) into a cementitious mortar. In particular, natural silica sand was replaced at three different volume fractions (10, 25 and 50%) with foamed end-of waste aggregates, as shown in Fig. 1 and listed in Table 1. Natural sand replacement with plastic aggregates reduced mortars density, as reported in Table 2.

**Table 1:** Lightweight mortars (LWMs) nomenclature and composition.

**Mortar**	***w/c***	**Silica sand (%)**	**LWA (%)**
**Reference**	0.50	100	–
**LWM10**	0.50	90	10
**LWM25**	0.50	75	25
**LWM50**	0.50	50	50

**Table 2:** Dry density of the investigated of the investigated LWMs.

**Dry density, *ρ***_**d**_**(g/cm**^**3**^**)**		**Δ*ρ***_**d**_**(%)**
**Reference**	2.143 ± 0.012	–
**LWM10**	1.981 ± 0.003	8
**LWM25**	1.802 ± 0.008	16
**LWM50**	1.374 ± 0.013	36

**Fig. 2:**
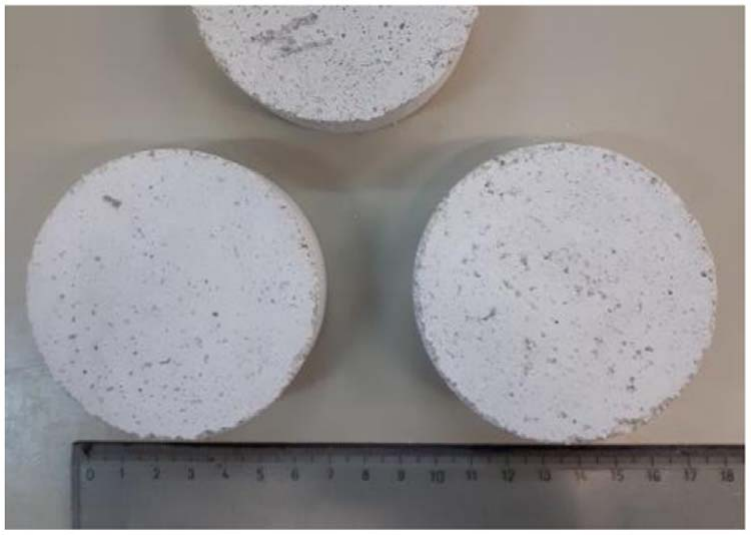
Example of cylindrical specimens used for the water vapour permeability test.

**Fig. 3:**
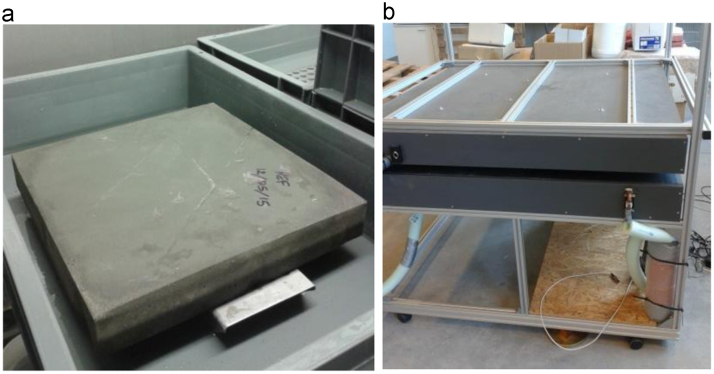
a) example of specimen used for the thermal conductivity test; b) GHP apparatus.

Moreover, the presence of artificial aggregates influenced also water absorption, water vapour permeability and thermal conductivity of the investigated mortars as reported in the research article [1]. Water absorption results are reported in Table 3 and such results were used to calculate mortars total open porosity (Table 4). In Table 5 are reported the values of the capillary water absorption coefficient, calculated according to EN 1015-18 [6]. Water vapour permeability was measured on cylindrical specimens (Fig. 2) according to EN 1015-19 [7]. Water vapour permeability and resistance are reported in Table 6, Table 7. Thermal conductivity measurements were performed using a GHP apparatus on mortars slab (Fig. 3); thermal conductivity of mortar specimens are listed in Table 8.

**Table 3:** Water absorption (*W*_ab_) of the investigated LWMs.

**Water absorption, *W***_**ab**_**(%)**		**Δ*W***_**ab**_**(%)**
**Reference**	8.02 ± 0.05	–
**LWM10**	8.83 ± 0.04	10
**LWM25**	9.60 ± 0.04	20
**LWM50**	18.74 ± 0.32	134

**Table 4:** Total open porosity of the investigated LWMs.

**Total open porosity, *P* (%)**		**Δ*P* (%)**
**Reference**	15.77 ± 0.24	–
**LWM10**	15.74 ± 0.33	0
**LWM25**	16.88 ± 0.09	7
**LWM50**	22.84 ± 0.57	45

**Table 5:** Capillary water absorption coefficient (*C*_w_) of the investigated LWMs.

**Capillary water ab. coeff., *C***_**w**_**[kg/(m**^**2**^**h**^**0.5**^**)]**		**Δ*C***_**w**_**(%)**
**Reference**	1.87 ± 0.02	–
**LWM10**	1.26 ± 0.08	32
**LWM25**	1.02 ± 0.09	45
**LWM50**	0.72 ± 0.08	61

**Table 6:** Water vapour permeability (*W*_*vp*_) of the investigated LWMs.

**Water vapour permeability, *W***_**vp**_**(kg/m s Pa)**		**Δ*W***_**vp**_**(%)**
**Reference**	2.97E-11 ± 1.39E-12	–
**LWM10**	3.60E-11 ± 1.73E-12	21
**LWM25**	3.97E-11 ± 1.45E-12	34
**LWM50**	5.88E-11 ± 3.32E-13	98

**Table 7:** Water vapour resistance factor (*μ*) of the investigated LWMs.

**Water vapour resistance, *μ***		**Δ*μ* (%)**
**Reference**	6.56 ± 0.31	–
**LWM10**	5.72 ± 0.17	13
**LWM25**	4.90 ± 0.18	25
**LWM50**	3.38 ±0.02	48

**Table 8:** Thermal conductivity of the investigated LWMs.

**Thermal conductivity, *λ* (W/m K)**		**Δ*λ* (%)**
**Reference**	1.408 ± 0.052	–
**LWM10**	1.365 ± 0.036	−3
**LWM25**	1.311 ± 0.027	−7
**LWM50**	1.266 ± 0.024	−10

## 2 Experimental design, materials and methods

### 2.1 Materials

Construction and building materials hygro-thermal and durability properties are key parameters in the life and maintenance of structures. The presence of plastic aggregates instead of natural quartz sand modifies mortar microstructure and, consequently, such properties. Table 1 presents the investigated mixtures prepared using an ordinary Portland cement (CEM I 42.5 N), standard quartz sand (0/2 mm) and plastic aggregates replacing natural quartz sand at three volume fractions (10, 25 and 50%). All the samples were prepared using a w/c ratio of 0.50 and sand/cement ratio of 3. Lightweight aggregates (LWAs) and silica sand were dry blended (Fig. 1) before the addition to the other mortar constituents (i.e. water and cement). Lightweight aggregates were prepared according the procedure described in [2]. The rough and porous surface of foamed aggregates is able to improve cementitious matrix/plastic aggregates interactions, which is one of the most important issue in the use of plastic fibers and/or aggregates in cementitious matrices [2], [4].

### 2.2 Methods

Mortars density was evaluated (three specimens for each composition) according to EN 12390-7 [5]. Table 2 summarizes mortars density and density variation, compared to the reference sample, of the investigated mixtures. As evident, at increasing natural aggregates substitution, a decrease of density was obtained.

Water absorption (*W*_ab_) was determined as the ratio between the amount of absorbed water (difference between the mass of the saturated and dry specimen) and the mass of the dry mortar specimen. Water absorption and water absorption variation, compared to the reference sample, are reported in Table 3. At increasing artificial aggregates content, an increase of water absorption occurred.

Total open porosity was calculated considering the ratio between the water absorption and specimen volume. Mortars porosity and porosity variation, compared to the reference sample, are reported in Table 4: at increasing plastic aggregates content, an increase of porosity was obtained.

The absorption of water due to the capillary rise was determined on three prismatic specimens for each mixture and the capillary water absorption coefficient (*C*_w_) was calculated according to EN 1015-18 [6]:

(1) $$C_{w}=0.1(M_{2}-M_{1})$$

where *M*_2_ and *M*_1_ are the mass of the specimen, in grams, after 90 min and 10 min of immersion, respectively. Prior to test, specimens were oven dried up to constant mass at 60 °C. Then, prismatic samples were immersed in deionized water for about 5 mm and the mass variation was measured. The results are reported in Table 5.

Water vapour permeability, *W*_*vp*_, of lightweight mortar samples was determined according EN 1015-19 [7] (Table 6):

(2) $$W_{\mathrm{vp}}=\frac{s}{A{∆}_{p}/(∆G/∆t)-R_{A}}$$

where *s, A* and *ΔG* are sample thickness, area and mass variation; *Δt* is the time interval; *Δp* is the gradient of water vapour tension between saturated solution and samples storing chamber and R_A_ is the resistance to water vapour diffusion in the air between the sample and the KNO_3_ saturated solution (0.048⋅10^9^ Pa m^2^ s/kg, for 10 mm of interspace). *W*_*vp*_ was measured on three flat cylindrical specimens (diameter of 75 mm and thickness of about 20 mm, Fig. 2), conditioned at RH = 50% and T = 20 °C, for each lightweight mortar. Specimens were sealed on glass containers, containing a saturated solution of KNO_3_, that provides a relative humidity of 93.2%, at 20 °C. The water vapour resistance factor (μ) was measured according to the following equation:

(3) $$\mu =\frac{\delta_{A}}{W_{\mathrm{vp}}}$$

where δ_A_ is air permeability (1.94⋅10^-10^ kg/(Pa m s)) in test conditions (20 °C and 50% RH) and *W*_*vp*_ is water vapour permeability. The results are summerised in Table 7.

Thermal conductivity measurements were carried out on mortar specimens of size equal to 30 × 30 × 5 cm^3^ (Fig. 3a), manufactured in wood molds. After 28 days of water curing, specimens were conditioned (25 °C and 50% RH) until constant mass before testing. Five tests were performed for each mortar sample to ensure the reproducibility. Thermal conductivity was measured using the guarded hot plate technique (GHP), defined in the ISO 8302 and specified in European standards EN 12664 [8] and EN 12667 [9]. The device used in this research (Fig. 3b) was designed, constructed and validated by Dubois and Lebeau [10].
